# 

**Published:** 1830-10

**Authors:** 


### MONTHLY LIST OF MEDICAL BOOKS.

A Treatise on the Venereal Diseases of the Eye. By WILLIAM LAWRENCE, F.R.S. &c. 8vo. pp. 337. Wilson, 1830.

A Short Tract on the Formation of Tumors, and the Peculiarities that are met with in the Structure of those that have become Cancerous; with their Mode of Treatment. By Sir EVERARD HOME, Bart. V.P.R.S. F.S.A. F.L.S. Serjeant Surgeon to the King, &c. 8vo. pp. 98. Plates. Longman, 1830.

A few Words on Quackery. By DEMOCRITUS, Junior. 8vo. pp. 23. Burgess and Hill, 1830.

Two Memoirs read before l'Academie Royale des Sciences, at Paris, on the successful Inhalation of diluted Chlorine, in the early Stages of Pulmonary Consumption, as a Remedy capable of prolonging Life, and of alleviating the distressing Symptoms in the more advanced Stages of that Complaint. With Cases, illustrating the Method of administering the Gas, and showing its beneficial Effects. Translated from the French of M. GANNAL, by WILLIAM HORATIO POTTER, M.R.I. Operative Chemist. 8vo. pp. 93. Callow and Wilson, 1830.

Practical Remarks on the Discrimination and successful Treatment of Spasmodic Stricture in the Colon, considered as an occasional Cause of habitual Confinement in the Bowels. Illustrated by Cases. By JOHN HOWSHIP, Surgeon to St. George's Infirmary, Teacher of Surgery, &c. 8vo. pp. 50. Burgess and Hill, London, 1830.

Anatomy of the Viscera, and Organs of Urine and Generation; arranged in three Tables. For the Use of Students. Burgess and Hill, 1830.

These Tables are now completed, and we again recommend them to anatomical students as very cheap and correct references. Their utility has already commanded a very extensive sale, and we doubt not that they will become more popular the more widely they are distributed.

The Laws relating to the Medical Profession; with an Account of the Rise and Progress of its various Orders. By J. W. WILLCOCK, Esq. Barrister at Law. 8vo pp. 450. Clarke, 1830.

*Communications received from* Dr. and Mr. D. GRIFFIN, Dr. BARRY, *&c.*

